# Nursing microtheory in the prevention of *delirium* in older adult in the intensive care unit*

**DOI:** 10.1590/1518-8345.6707.4071

**Published:** 2023-12-04

**Authors:** Sandra da Silva Kinalski, Margrid Beuter, Eliane Raquel Rieth Benetti, Marinês Tambara Leite, Larissa Venturini, Marcos Antônio Gomes Brandão

**Affiliations:** 1 Universidade Federal de Santa Maria, Santa Maria, RS, Brasil.; 2 Universidade Federal de Santa Maria, Departamento de Ciências da Saúde, Palmeira das Missões, RS, Brasil.; 3 Universidade Federal de Santa Maria, Palmeira das Missões, RS, Brasil.; 4 Universidade Federal de Santa Maria, Hospital Universitário de Santa Maria, Santa Maria, RS, Brasil.; 5 Universidade Federal do Rio de Janeiro, Rio de Janeiro, RJ, Brasil.; 6 Becario del Conselho Nacional de Desenvolvimento Científico e Tecnológico (CNPq), Brasil.

**Keywords:** Nursing, Aged, Delirium, Nursing Theory, Intensive Care Units, Nursing Care, Enfermería, Anciano, Delirio, Teoría de Enfermería, Unidades de Cuidados Intensivos, Atención de Enfermería, Enfermagem, Idoso, Delirium, Teoria de Enfermagem, Unidade de Terapia Intensiva, Cuidados de Enfermagem

## Abstract

**Objective:**

to describe a microtheory for nursing care in the prevention of *delirium* in older adult in the intensive care unit.

**Method:**

prescriptive theoretical research, based on substruction. Roy’s Adaptation Model constructs were deduced and data from the phenomenon of nursing care in the prevention of *delirium* in older adult in intensive care were induced, based on an integrative literature review.

**Results:**

the microtheory has a theoretical and operational system and a model of care. In the theoretical system, Roy’s focal and contextual stimulus constructs were used. From them, the concepts of focal and contextual care and the variable adaptive response to prevention were elaborated. From the relational statements, four axioms, two postulates, eight propositions and an epistemic assumption were elaborated.

**Two empirical indicators were established in the operating system:**

the Confusion Assessment Method for Intensive Care Units and the demographic/clinical history of the older adult. Subsequently, two transformational statements, four hypotheses and the model of care represented in figure were produced.

**Conclusion:**

the microtheory produced prescribes care in the prevention of *delirium* in older adult in intensive care, through a construct of interest to nursing, and allows interceptions for the development of instruments that guide nursing activities.

## Abstract (es)

**Objetivo:**

describir una microteoría para cuidados de enfermería en la prevención del delirio en personas mayores en la unidad de cuidados intensivos.

**Método:**

investigación teórica prescriptiva, basada en la subconstrucción. Se dedujeron constructos del Modelo de Adaptación de Roy y se indujeron datos del fenómeno de cuidados de enfermería en la prevención del *delirium* en personas mayores en cuidados intensivos, a partir de una revisión integrativa de la literatura.

**Resultados:**

la microteoría posee sistema teórico, operativo y modelo de cuidado. En el sistema teórico se utilizaron los constructos estímulo focal y contextual de Roy. A partir de ellos, se elaboraron los conceptos de cuidado focal y contextual y la variable respuesta adaptativa a la prevención. De las declaraciones relacionales se elaboraron cuatro axiomas, dos postulados, ocho proposiciones y una presuposición epistémica.

**En el sistema operativo se establecieron dos indicadores empíricos:**

el *Confusion Assessment Method for Intensive Care Units* y el historial demográfico/clínico de las personas mayores. Posteriormente, se produjeron dos declaraciones transformacionales, cuatro hipótesis y el modelo de cuidado representado en figura.

**Conclusión:**

la microteoría producida prescribe cuidados en la prevención del *delirium* en personas mayores en cuidados intensivos, por un constructo de interés para la enfermería, y permite intervenciones en el desarrollo de instrumentos que guían la actuación de la enfermería.

## Abstract (pt)

**Objetivo:**

descrever uma microteoria para cuidados de enfermagem na prevenção do *delirium* em pessoas idosas na unidade de terapia intensiva.

**Método:**

pesquisa teórica prescritiva, baseada na substrução. Foram deduzidos construtos do Modelo de Adaptação de Roy e induzidos dados do fenômeno dos cuidados de enfermagem na prevenção do *delirium* em pessoas idosas na terapia intensiva, a partir de revisão integrativa da literatura.

**Resultados:**

a microteoria possui sistema teórico, operacional e modelo de cuidado. No sistema teórico utilizaram-se os construtos estímulo focal e contextual de Roy. A partir deles elaboraram-se os conceitos de cuidado focal, contextual e a variável resposta adaptativa à prevenção. Das declarações relacionais elaboraram-se quatro axiomas, dois postulados, oito proposições e um pressuposto epistêmico. No sistema operacional, estabeleceram-se dois indicadores empíricos: o *Confusion Assessment Method for Intensive Care Units* e o histórico demográfico/clínico das pessoas idosas. Posteriormente, produziram-se duas declarações transformacionais, quatro hipóteses e o modelo de cuidado representado em figura.

**Conclusão:**

a microteoria produzida prescreve cuidados na prevenção do *delirium* em pessoas idosas na terapia intensiva, por um construto de interesse para a enfermagem, além de permitir interceptações ao desenvolvimento de instrumentos que norteiam a atuação da enfermagem

**Highlights:**

**(1)** It prescribes care “for” and “in” the prevention of *delirium* in older adult in the ICU.

**(2)** Microtheory developed by theoretical substruction for use in clinical practice.

**(3)** Tool with a philosophical perspective that guides nursing care.

**(4)** Empirical indicators are important to evaluate the effectiveness of interventions.

## Introduction

Delirium is a cognitive syndrome with a prevalence of 9 to 32 *%* in hospitalized patients ^(^ 1 ^)^. It is a frequent disorder among older adult hospitalized in an intensive care unit (ICU) ^(^ 2 ^)^, associated with the presence of factors related to this environment and their routines ^(^ 3 ^)^. Still, when it comes to older adult with risk factors such as: advanced age, frailty, cognitive deficit, multiple comorbidities, alcoholism, smoking, previous coma, trauma and dementia, the probabilities of *delirium* occurrence increase ^(^ 4 ^-^ 6 ^)^. 

Given its relevance, in 2017 the Intensive Care Delirium Research Agenda was published *,* the result of a multinational and interprofessional perspective, presenting the research needs on *delirium* ^(^ 7 ^)^. Among the main areas of study recommended for the next 10 years is the development of new models to refine the phenotyping of *delirium*, that is, to recognize etiological elements that may be associated with it. 

Although doctors can contribute to the investigation of causality based on pathophysiological models, this perspective of phenotyping/identification of etiological factors can be limiting for nursing practice. Thus, the perspective of the nursing diagnosis focus was used to guide the human response and the care directed by interventions of a preventive nature. Studies on nursing care in the prevention of *delirium* in the ICU are carried out, however their contributions are diverse, especially when referring to older adults ^(^ 6 ^-^ 8 ^)^. 

Theories can provide explanatory, predictive, or prescriptive models that are useful when evidence seems scattered. The paradigm of practice guided by nursing theory has played a prestigious role throughout the discipline’s history, although it may have less interest depending on the group of researchers ^(^ 9 ^-^ 10 ^)^. Furthermore, nursing theories, although they are disciplinary constructions, must be useful and compatible with use in the multidisciplinary and multidisciplinary context of health ^(^ 11 ^)^. Therefore, microtheories created in the discipline of nursing can produce conceptualizations, theoretical statements and models based on phenomena of multidisciplinary interest, such as *delirium*. 

Among the theoretical levels, microtheories offer a practical way for nurses to connect the discipline’s philosophical perspectives with the real world, by prescribing interventions for nursing practice ^(^ 12 ^)^. They are characterized by producing specific instructions for practice and being the most applicable of all theories, as they are at a lower level of abstraction, closer to the empirical level of phenomena, when compared to the great nursing theories ^(^ 13 ^)^. Thus, microtheories have a direct impact on nursing care, given their limited time, place and situation, they are concerned with more specific areas of knowledge and are interdependent ^(^ 14 ^)^. 

In this article, it is assumed that a microtheory can provide an explanatory model of phenotyping/etiologies of interest to nursing, mechanisms and other conditions associated with *delirium* in older adult in the ICU, based on the scientific evidence in the literature for this population. Therefore, with theorization, when combining data from the integrative literature review and Callista Roy’s Adaptation Model (RAM), it is inferred that both the nurse’s clinical judgment and the choice of actions contribute to intervene on the phenomenon. Thus, the objective is to describe a microtheory for nursing care in the prevention of *delirium* in older adult in the intensive care unit. 

## Method

### Study design

This is a theoretical study, in which a microtheory was developed, of the prescriptive type, of a basic nature ^(^ 15 ^)^. 

### Data collect

To elaborate this microtheory, theoretical data from RAM and the integrative review were combined, through deductive and inductive guidelines for approaching theoretical thinking. In the deductive orientation, the theoretical substruction ^(^ 16 ^)^ was used as a strategy to produce congruence between the theory of RAM and an operational system generated in the present microtheory. The RAM theory, in its central aspects, involves the tendency to look at people as open systems, which maintain continuous interaction with the environment, being exposed to different stimuli that generate a process of dealing with challenges, capable of resulting in adaptive responses or ineffective ^(^ 17 ^)^. The theoretical substruction constituted in this microtheory the theoretical and operational systems. 

In the inductive orientation, the scientific literature synthesis approach was used, through the integrative review ^(^ 18 ^)^. The review was used to find elements and achieve homogeneity of data on nursing care for the prevention of *delirium* in older adult in the ICU ^(^ 19 ^)^. 

### Data analysis

In the first and second stages, the identification and selection of RAM constructs was carried out. Constructs are abstract notions that can be partially defined ^(^ 16 ^)^. Initially, there was an in-depth familiarization with the RAM ^(^ 17 ^)^, through careful reading. After the RAM content was examined, the focal stimulus and contextual stimulus constructs were selected for theoretical substruction. 

Next, an integrative review of the literature on nursing care for the prevention of *delirium* in older adult in the ICU was carried out. The literature synthesis followed the phases: 1 - identification of the problem; 2 - search in the literature; 3 - data evaluation; 4 - data analysis; 5 - presentation ^(^ 18 ^-^ 19 ^)^. Thus, search strategies were developed for each database, combining different descriptors and keywords. The search took place in the Federated Academic Community (CAFe) of the Coordination for the Improvement of Higher Education Personnel (CAPES), at the US National Library of Medicine National Institutes of Health (PubMed), Scopus *,* Cumulative Index to Nursing and Allied Health Literature (CINAHL), Latin American and Caribbean Literature in Health Sciences (LILACS) and the Web of Science, without using a time frame. 

A total of 17,126 studies were identified in PubMed (2,368), Scopus (8,553), CINAHL (908), LILACS (486), Web of Science (4,433). Of these, 16,986 were duplicate studies or from non-primary sources and were therefore removed. Inclusion and exclusion criteria were applied, resulting in 80 complete texts. After reading in full, based on the answer to the review question, the final sample totaled 51 articles (12 in PubMed, 16 in Scopus, 11 in CINAHL, 5 in LILACS and 7 in Web of Science). From a theoretical point of view, the evidence from the integrative review contributed to the development of assumptions in the theoretical system and to the inclusion and organization of elements of the operating system of this microtheory.

Microtheory ^(^ 16 ^)^ was developed in four steps: 1 - identify and isolate the main constructs and concepts under study; 2 - specify relationships between concepts; 3 - hierarchically order the concepts by level of abstraction; 4 - describe the diagram of the relationships between the variables. Subsequently, microtheory modeling was carried out, theoretical, coherent and interconnected knowledge about the phenomenon of nursing care in the prevention of *delirium* in older adult in the ICU, resulting in a model, represented by a graphic figure. 

**Figure 1 -:**
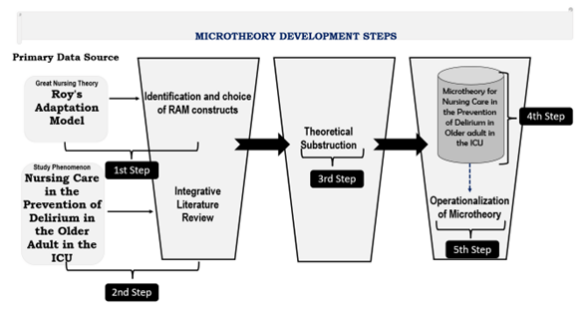
Figure scheme to represent the development of MiTCare-DEP ^*^

^*^MiTCare-DEP = Micro Theory of care for delirium in older adult; ^†^ICU = Intensive Care Unit; ^‡^RAM = Roy’s Adaptation Model

The microtheory development process is shown in Figure 1. 

### Ethical aspects

The present research, of a theoretical nature, respected the copyright of the publications included in the study.

## Results

The micro theory of nursing named Micro Theory of Care for Delirium in Elderly Patient (MiTCare-DEP) aims to indicate interventions that help nursing care in the prevention of *delirium* in older adult in the ICU. Microtheory has the theoretical system, the operating system, the relational statements between the variables chosen to compose each system ^(^ 16 ^)^ and a theoretical model. 

### Theoretical system

The theoretical system includes as elements: constructs, concepts, subconcepts (variables) and empirical indicators; and as relational statements: axioms, postulates and propositions ^(^ 16 ^)^. Although not included in the original methodological proposal of substruction, six assumptions were elaborated ^(^ 14 ^)^, through which the theoretical description begins. 

The assumptions of a theory are characterized by information taken as true and are based on what theorists consider evidence, based on values and beliefs ^(^ 14 ^)^. Because MiTCare-DEP is a scientific theory, based on the synthesis of empirical evidence, its theoretical assumptions have a lower level of abstraction, therefore, its values and beliefs are not purely philosophical. They were developed in order to build a structural foundation of the phenomenon under study, namely:

1. The ICU is permeated with specificities in its structure and care routines, which are stimuli for triggering delirium in the older adult.
2. The aging of the older adult and, when present, cognitive impairment, can hinder the development of coping mechanisms to prevent *delirium*.
3. Older adult hospitalized in ICUs need systematized and organized nursing care for the prevention of *delirium*.
4. Nursing care in the prevention of *delirium* must be continuous and present throughout the period of hospitalization of older adult in the ICU.
5. Focal carrated into nursing care to prevent delirium in older adult hospitalized in the ICU.
6. Aspects of the demographic/clinical history and health problems of the older adult, such as: age, smoking, alcoholism, cognitive impairment and dementia constitute stimuli for non-adaptive responses to the prevention of delirium by the elderly in the ICU.

Two major blunt and delimited theoretical constructs were deduced from Callista RAM, to describe and explain the phenomenon based on microtheory for nursing. Focal stimuli are events closely related to the occurrence of the situation, which immediately confront people and constitute the greatest degree of change, triggering responses that can be adaptable or ineffective ^(^ 17 ^)^. Contextual stimuli are all the environmental factors that are presented to the person, they are not the center of attention and, even so, they influence the way the person deals with their focal stimulus ^(^ 17 ^)^. 

In the conceptual development of MiTCare-DEP, properties brought by constructs identified in RAM ^(^ 17 ^)^ and induced by literature data were used. In order to explain the nursing care phenomenon in the prevention of *delirium* in older adult in the ICU, the subsumed concepts were: focal care and contextual care. 

Focal care refers to a set of interventions, presented in Figure 2, consisting of nursing activities in the prevention of *delirium* in older adult in the ICU, which act as an external stimulus and, immediately, trigger an effective response in the person being cared for prevention of *delirium*, closely related to the particularities and completeness of the older adult. Contextual care, on the other hand, refers to a set of interventions, presented in Figure 3, consisting of nursing activities in the prevention of *delirium* in the ICU, which act as contextual stimuli of the environment and contribute to the effective response in the prevention of *delirium* by the older adult, closely related to the specificities of intensive care. 

**Figure 2 -:** Constitutive definitions of the focal care concept of MiTCare-DEP

Focal Care	Interventions
Carrying out guidance activities	Promote time, place and character orientation activities (>3x/day). Carry out activities with calendars, clocks, cell phones and radios. Dialogue with older adult about past experiences and current activities.
Sensory Interventions for Visual and Hearing Impairment	Promote visual and auditory stimulation through the use of visual and hearing aids. Take an approach that facilitates visual and auditory contact. Promote assertive verbal communication, providing material adapted for visual impairments. Protect the cornea during sedation, preventing dehydration.
Sleep pattern maintenance	Provide indirect lighting, especially at night. Provide an eye mask and/or ear plugs. Adjust the medication regimen, avoiding dosing between 00:00 and 05:00 hours. Provide music therapy sessions. Reduce environmental noise. Perform pain control. Do not perform elective procedures at night. Teach and encourage deep breathing and relaxation exercises before bed. Offer massage on the feet and thoracic and lumbar regions.
Early mobilization	Promote patient mobilization in or out of bed, within 24 to 48 hours after admission to the ICU. Plan mobility schedule with range of motion exercises, active, passive and walking when possible, three times a day.
Therapeutic communication	Maintain clear and open communication with the older adult and encourage their emotional expression. Use face-to-face communication with eye contact. Use the patient’s name during communication and introduce yourself to the patient when first contact occurs. Allow them to express their thoughts and feelings. Use a communication card, WordPad or pen and paper for those undergoing endotracheal intubation or tracheostomy.
Music therapy	Provide moments of music therapy through MP3 players, as well as headphones. This action can be collective or individual.
Hydration and nutrition	Evaluate the nutritional status of the elderly on admission to the ICU, using scales such as: Geriatric Nutritional Risk Index (GNRI), Prognostic Nutrition Index (PNI), Controlling method Nutritional Status (CONUT). Ensure adequate nutrition and hydration.
Cognitive stimulation	Care aimed at stimulating cognitive function, for this, materials adapted to the needs of the older adult should be used, such as large keyboards, clocks with larger displays and books with large letters. Daily activities are recommended, at least three times a day, to stimulate memory, such as discussing current events in family life and remembering past events. To carry out the activities, the use of books, magazines and the presence of family members in the ICU is associated. Also, they can be used as stimulating activities, playing cards, word search games, crossword puzzles (daily duration of five minutes). For patients undergoing endotracheal intubation or tracheostomy, a communication card, pen, paper or WordPad can be used to establish communication and help with activities.
Night nursing care	Schedule so that most care is performed during the day. Group the care that is performed at night for specific times, avoiding repeated sleep interruptions.
Mechanical containment and indwelling catheters	Reduce the use of mechanical restraint and permanent catheters whenever possible, in order to avoid the physical restriction of the older adult in bed.
Family training on delirium	Train the patient’s family about delirium and its complications: definition, symptoms, etiology, negative effects, prevention of delirium. Education strategies such as video, folders, pamphlets, checklist.
Family participation in care	Stimulate the active participation of family members and patient companions, as emotional and affective support. Also, train them to participate in cognitive stimulation, guidance, hygiene and comfort activities for the elderly, under the supervision of the ICU nursing team.

**Figure 3 -:** Constitutive definitions of the MiTCare-DEP concept of contextual care

Contextual Care	Interventions
Provide materials such as: clocks, calendars, cell phones, books, magazines, radios, televisions, whiteboards, pens, papers and personal belongings of the patient	Provide and make available materials that can be used in temporal and spatial orientation, cognitive stimulation and communication with the older adult. Also, encourage their involvement in the proposed activities.
Reduce artificial lighting at night	Provide indirect lighting at night to maintain sleep patterns.
Reduce the environmental noise in the ICU	Monitor and maintain adequate noise volume levels in the ICU.
Promote privacy	Maintain, whenever possible, individual beds, using curtains or screens, with a view to the well-being of the patient and the performance of care.
Adjust monitor and machine alarms	Set monitors and machines to night mode, which lowers volume and dims displays, reducing noise and promoting sleep.
Provide natural light in the environment	Provide physical structure that allows natural light to the patient, promoting day/night orientation.
Control the ambient temperature	Maintain a pleasant ICU room temperature.
Flexibility in visiting hours in the ICU	Allow and encourage the visit of family members or companions who have an emotional relationship with the older adult.

After defining the concepts, the variables related to the dimensions of the study phenomenon are described. Variables are subconcepts subsumed from general concepts, used to order the phenomenon according to some property ^(^ 17 ^)^ . The study variables (subconcepts) must be epistemically linked to the concepts identified in the microtheory, thus, the variable - adaptive response to prevention - was subsumed from the general concepts. 

The adaptive response to prevention is the absence of *delirium* in the older adult in the ICU, as a condition resulting from the action of focal care and contextual care. This variable measures the assertiveness of nursing care in preventing *delirium* in older adult, based on the relationships between concepts, within the specific context of the ICU. 

Still, regarding the theoretical system of microtheory, the constructs, concepts and variables must be linked by relational statements, which affirm a relationship of some kind, called axioms, propositions, postulates and epistemic presuppositions ^(^ 16 ^)^. The direct and non-linear relationship between the constructs determined the axioms of this microtheory, which are based on the incorporated prescriptive power of the theoretical framework and articulate nursing care in the prevention of delirium with the older adult and the ICU environment. Four axiomatic statements for the constructs are presented:

1. There is a dynamic and non-linear interaction between focal stimuli and contextual stimuli, which form higher levels of complex organization of nursing care in the prevention of *delirium* in the older adult in the ICU.
2. Nursing care in the prevention of *delirium* in the older adult in the ICU is formed by a set of multifactorial influences of focal stimuli and contextual stimuli, which interact in a non-linear and complex way, capable of triggering coping processes in the older adult in the ICU.
3. Contextual stimuli represent the gathering of a set of non-linear, multifaceted influences, with complex interaction present in the ICU, which are presented to the older adult, which are not the person’s center of attention, but contribute to the effects of focal stimuli.
4. The focal stimulus represents the gathering of a set of influences arising from care interventions in the prevention of *delirium* in the ICU, which immediately confront the older adult, being potentiated by contextual stimuli.

Following the constitution of the theoretical system of microtheory, links between theoretical constructs and concepts were established ^(^ 16 ^)^ . Thus, two postulates were identified for MiTCare-DEP:

The concept of focal care represents the main aspects of focal stimuli characterized by nursing care in the prevention of *delirium* that immediately confront older adult in the ICU.
Contextual care represents the main aspects of contextual stimuli, which are important nursing care in the prevention of *delirium* directed to the ICU environment.

The relationship established between the concepts of the microtheory exemplifies how nursing care, in the prevention of *delirium* in older adult in the ICU, must be effectively implemented in clinical practice. Thus, it is possible to understand that the nursing practice is conditioned to the implementation of these concepts and their relationships to prescribe interventions to the phenomenon. The propositions represent the direct, bilateral and non-linear relationship between the concepts ^(^ 16 ^)^. Thus, eight propositions were elaborated in the theoretical system:

1. Nursing care in the prevention of *delirium* in older adult in the ICU is a multifactorial construct, with an effect enhanced by the association of the constituent units of the concepts of focal care and contextual care.
2. If, through contextual care, materials are made available for the implementation of cognitive stimulation activities for the older adult in the ICU, the effect of focal care aimed at guiding the older adult influences the prevention of *delirium*.
3. When contextual care does not offer interventions to reduce lighting at night, reduce environmental noise by adjusting the alarms of ICU equipment, promoting privacy for the older adult, controlling temperature and promoting natural light in the environment of the In the ICU, focal care may not generate an adaptive response to the prevention of *delirium* in the older adult in the ICU.
4. The flexibility of visiting hours in the ICU, the family’s orientation about *delirium* and their participation in the care of the older adult influence the adaptive response to the prevention of *delirium* in the older adult in the ICU.
5. Carrying out sensory interventions for visual and hearing impairments, promoting the maintenance of sleep patterns, offering therapeutic communication and music therapy, adapting equipment such as large keyboards, watches with larger displays and books with large letters, are care practices that share the influences of the contextual care and influence each other.
6. If the older adult’s hydration and nutrition are not adequate, then there may be a risk that focal care and contextual care may not be effective enough for the adaptive response to the prevention of delirium *in* the older adult in the ICU.
7. Nursing care during the night, early mobilization, mechanical restraint and the use of permanent catheters by the older adult are interventions that are not influenced by contextual care, but influence one another in the adaptive response to the prevention of *delirium* in older adult in the ICU.
8. If the actions of focal care and contextual care are in balance, prevention of *delirium* as an adaptive response of the older adult in the ICU is achieved.

Regarding the links between the concepts and the subconcept, these are described in relational statements called epistemic assumptions ^(^ 16 ^)^. The epistemic assumption elaborated for MiTCare-DEP is: the adaptive response to prevention represents the final product of a set of non-linear, multifaceted, complex interaction influences, subject to independent nursing care, categorized in the constitutive definitions of focal care and contextual care. 

### Operational system

The operationalization phase of microtheory converts ideas and relationships produced in the previous stage into observable components, applicable in clinical practice and subject to confirmation/rebuttal ^(^ 20 ^)^. The operating system includes empirical indicators, scores, values and measures of the study variables ^(^ 16 ^)^. In this microtheory, the use of two empirical indicators is proposed to measure the adaptive response to prevention variable: the Confusion Assessment Method for Intensive Care Units (CAM-ICU) ^(^ 21 ^)^ and the demographic/clinical history of older adult (alcoholism, smoking, prior cognitive impairment and dementia). 

In order to establish the links between the theoretical system and the operational system, transformational statements were developed, which represent the relationships between variables and empirical indicators ^(^ 16 ^)^. MiTCare-DEP presents the following transformational statements *:*

1. The CAM-ICU ^(^ 21 ^)^ is the empirical indicator to assess the adaptive response to prevention to verify the presence of *delirium* in the older adult in the ICU.
2. Demographic and clinical factors are empirical indicators to stratify the adaptive response to prevention by the older adult in the ICU.

Among the empirical indicators, relational statements called hypotheses were proposed to test the propositions of the microtheory. The hypotheses (H) generated are subject to quantitative analysis, are congruent with the totality, and represent an inferential and correlational descriptive view ( Figure 2): 

H1 - Demographic and clinical history factors (alcoholism, smoking, previous cognitive impairment and dementia) will be significantly associated with the total CAM-ICU score ^(^ 21 ^)^ . 

H2 - The relationship between alcoholism, smoking, previous cognitive impairment and dementia will be evaluated by the CAM-ICU ^(^ 21 ^)^ , after identifying demographic and clinical history factors. 

H3 - Prior cognitive impairment and dementia will be significantly associated with more errors by older adult in the CAM-ICU result ^(^ 21 ^)^. 

H4 - Older adult will have more correct answers on the CAM-ICU ^(^ 21 ^)^ in the absence of all the demographic and clinical factors selected and considered as risk factors (alcoholism, smoking, previous cognitive impairment and dementia). 

**Figure 4 -:**
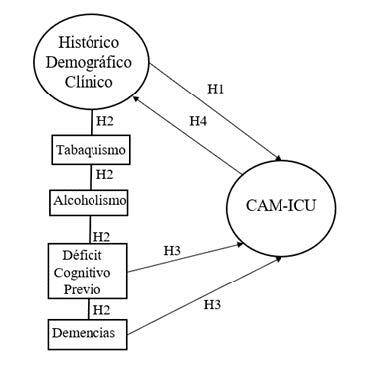
Diagram of Hypothesized Relational Statements

^*^H = Hypotheses; ^†^CAM-ICU = *Confusion Assessment Method for Intensive Care Units*

A diagram relating the theory’s operating system assumptions is shown in Figure 4. 

With regard to theoretical limits, in terms of abstraction, MiTCare-DEP is a micro theory or micro range theory ^(^ 22 ^)^, in which its low level of abstraction prescribes recommendations for the practice of nursing care in the prevention of *delirium* in people older adult in the ICU. As for the scope, the microtheory is limited: (1) by the phenomenon of *delirium*; (2) by the population affected by the phenomenon, the older adult; (3) by the ICU care context; and (4) the nature of the theoretical prescription of preventive actions through nursing care. 

### MiTCare-DEP care model

**Figure 5 -:**
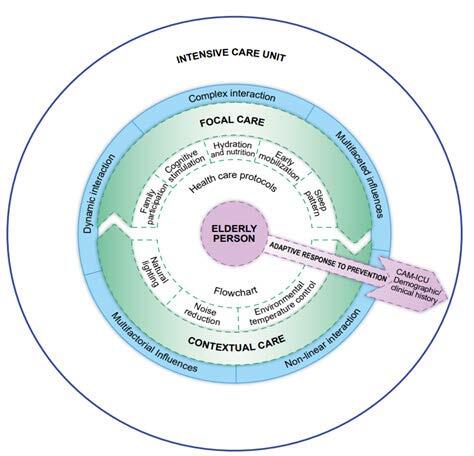
MiTCare-DEP care model

^*^MiTCare-DEP = Micro Theory of Care for Delirium in Older adult; ^†^CAM-ICU = Confusion Assessment Method for Intensive Care Units

Once the elements of the theoretical and operational system were developed, a nursing care model was designed to prevent *delirium* in the older adult in the ICU, shown in Figure 5, which graphically illustrates the theoretical relationships between the theoretical constructs of RAM and the phenomenon of this study, such as also the possibilities of implementing MiTCare-DEP in nursing practice. 

MiTCare-DEP, based on the substruction of the constructs focal stimuli and contextual stimuli, indicates that there is a dynamic, non-linear and complex interaction between these stimuli, requiring high levels of organization of nursing care. The interaction between focal stimuli and contextual stimuli support that nursing care in the prevention of *delirium* in the older adult in the ICU is formed by a set of multifactorial influences, which interact in a dynamic, non-linear and complex way, capable of stimulating the adaptive response in the older adult in ICU, preventing *delirium*. 

Focal care and contextual care should be implemented in the ICU so that nursing care in the prevention of *delirium* is the necessary stimulus for the older adult to avoid triggering *delirium*. Focal care and contextual care are sets of interventions consisting of nursing activities in the prevention of *delirium* in older adult in the ICU, which bring together the multifaceted influences necessary for the prevention of *delirium*. 

In this context, nursing care in the prevention of *delirium* in the older adult in the ICU is understood as a multifactorial construct, which, through the association of focal care and contextual care, allows an adaptive response to the prevention of *delirium* by the older adult. 

To verify the effectiveness of focal care and contextual care, it is necessary to evaluate the variable adaptive response to the prevention of *delirium* by the older adult in the ICU. Thus, measurement using the CAM-ICU ^(^ 21 ^)^ and the stratification of some factors of the demographic and clinical history of the older adult in the ICU is suggested. The *Confusion Assessment Method* (CAM) and its ICU variable are widely used in geriatrics ^(^ 23 ^)^. 

It is inferred that nursing should implement MiTCare-DEP in practice, through care management instruments, care protocol and flowchart, as guides for the implementation of the nursing process. Focal care and contextual care should be implemented throughout the entire period of hospitalization of the older adult in the ICU, due to the benefits they may bring with the prevention of *delirium*. 

In this way, nursing practice operationalized by focal care and contextual care for older adult in the ICU is a philosophy of nursing care that emphasizes nursing care “for” and “in” the prevention of delirium, applied to older adult *and* to the ICU environment. The prevention of *delirium* in older adult in the ICU requires that nursing work go beyond the mechanized approach, tied to rigid care routines, unifying critical patient care, the professional development of the nursing team and the advancement of disciplinary knowledge in the area. 

## Discussion

The core of the MiTCare-DEP proposal is to develop a microtheory, of the prescriptive type ^(^ 24 ^)^, for nursing care in the prevention of *delirium* in older adult in the ICU, supported by RAM constructs. Thus, for the production of a theory, the selection of constructs should reflect the topic or area of greatest interest to the researcher, based on what is most critical, which is convenient for the process of theoretical development, therefore, it is opportune to select associated constructs to the study phenomenon ^(^ 25 ^)^. 

Using the meaning of the focal stimulus and contextual stimulus constructs was useful to produce congruence between the abstract elements of the RAM with empirical conditions that provoke the stimuli, in a causal relationship. It can be a focal stimulus when a person turns quickly, when a loud noise comes from behind (external stimulus), or is irritated by a ringing in the ear (internal stimulus). The person concentrates on the focal stimulus and expends energy to deal with it, that is, from the stimulus, the person tries to find its source to decide relatively how to deal with it ^(^ 17 ^)^. 

Analogously to the RAM focal stimulus construct, the MiTCare-DEP focal care concept is closely related to the particularities and completeness of the older adult, as the constitutive definitions of this concept are composed of activities directed at the person being cared for, which act as an external stimulus and immediately trigger an effective response to prevent *delirium*. Thus, nursing care should focus on health promotion, disease prevention, health recovery and rehabilitation, focusing on the patient and his biopsychosocial-spiritual needs ^(^ 26 ^)^. With regard to gerontological nursing care, this must be based on the integrality and autonomy of the older adult human being ^(^ 27 ^)^. 

Regarding the contextual stimulus construct, to exemplify it, the common experience with the climate can be highlighted. It is known that it is not the climatic temperature alone that makes us react to heat or cold. When high humidity is associated with high temperatures, the heat is less tolerable, and when a cool wind is combined with cold temperatures, the cold is more affected. Thus, it is understood that while more attention is given to the focal stimulus, the contextual stimuli are those that can also be identified as influencing the situation ^(^ 17 ^)^. 

In the same sense, in MiTCare-DEP, the contextual care concept, subsumed by the contextual stimulus construct, is composed of a set of interventions, related to the specificities of the context of intensive care, which act as contextual stimuli of the environment. Thus, it is understood that for interventions to act as stimuli in the older adult, nursing care must be focused on the older adult and also on the environment (ICU) in which they are inserted. The care receiver can be a person, a family, a community or a society, and each one of them is considered, by the nurse, as a holistic adaptive system ^(^ 17 ^)^. 

This statement is due to the presence of modifiable risk factors present in the ICUs, which contribute to the occurrence of *delirium* , such as: noisy environment, artificial lighting, change of habits, sleep deprivation, social isolation, rotation of professionals, physical restraint, pain, sedoanalgesia, invasive devices – tubes, probes and mechanical ventilation ^(^ 3 ^)^. 

Still, the unknown environment, distance from family and friends become facilitating factors for the emergence of complications in older adult hospitalized in the ICU. These factors trigger physical and psychological impacts, which often go beyond what the older adult has already experienced, making them more prone to triggering *delirium* ^(^ 28 ^)^. Thus, the hospitalization of the older adult in the ICU requires the promotion of strategies for the prevention of *delirium* by the nursing team, in order to encourage coping and adaptation in the intensive environment ^(^ 29 ^)^. 

In this context, focal care and contextual care, based on the theoretical-scientific basis of MiTCare-DEP, have the potential to leverage the conditions of the older adult to achieve an adaptive response to prevention. Thus, the importance of preventing *delirium* in the older adult is reinforced by the fact that this condition increases the duration of mechanical ventilation, decreases functionality and post-discharge quality of life of the older adult who was hospitalized in an ICU ^(^ 27 ^)^. *Delirium* is a serious condition that has become part of the patient safety agenda ^(^ 30 ^)^ and has been identified *as* an indicator of health quality for the older adult ^(^ 2 ^)^. 

Faced with the notoriety of the complications that affect older adult in the event of *delirium*, it is important for health professionals to develop strategies to prevent the syndrome, through institutional protocols that guide safe and quality care ^(^ 31 ^)^. In this context, there is an urgent need to implement the MiTCare-DEP care instruments in intensive nursing practice, such as the clinical protocol and the flowchart. 

Still, given the magnitude of the problem imposed on older adult in the ICU by *delirium*, the use of care interventions focused on the multifactorial nature of the syndrome and the multidimensionality of the older adult for prevention stands out as a challenge to the nursing team ^(^ 32 ^)^. For this, it is necessary for professionals to understand the relationships that are established between the older adult, the ICU and *delirium*, described by MiTCare-DEP. 

In this sense, the microtheory advocates that there is a dynamic and non-linear interaction between focal stimuli and contextual stimuli, which form higher levels of complex organization of nursing care in the prevention of delirium in older adult in the ICU. In view of this, nursing care focused on preventing *delirium* has proven to be the best alternative for reducing the incidence of *delirium* in the ICU ^(^ 3 ^)^. Non-pharmacological interventions are shown to be the cornerstone for the management of *delirium* ^(^ 33 ^)^. 

We believe that the congruence developed between the constructs of focal and contextual stimuli with etiologies or risk factors for *delirium* may have produced a phenotyping model, which was identified as one of the relevant items for the research agenda on the subject ^(^ 7 ^)^. By using nursing care as a focus, MiTCare-DEP incorporated etiologies of professional interest to nurses to produce association relationships that generate different phenotypes modifiable by nursing interventions, such as, for example, *delirium* associated with noise (focal stimulus) or *delirium* associated with excessive heat (contextual). 

Although the idea of isolated etiologies in focal and contextual stimuli has theoretical and operational relevance, the theorized phenomenon has a multifactorial origin, with an effect enhanced by the association of the constitutive units of the concepts of focal care and contextual care. Therefore, the phenotypic reduction to a single etiological factor must be a rare condition, and it is expected that the phenotypic models are a human response to related multifactors.

The verification of empirical indicators and values of the operating system of the microtheory can be obtained by using instruments such as the CAM-ICU ^(^ 21 ^)^ and the demographic/clinical history of the older adult (alcoholism, smoking, previous cognitive impairment and dementia). These empirical indicators can predict the effectiveness of care, through reliable and valid measures, which produce scores (values), which ultimately represent the operationalization of the variable adaptive response to prevention ^(^ 16 ^)^. 

By exploring the components of the conceptual structure of the microtheory, it is proposed that the CAM-ICU ^(^ 21 ^)^ and some demographic/clinical factors represent an operational system to assess the adaptive response to prevention of older adult in the ICU. This is due to the high confirmatory power for *delirium* in critically ill patients on the scale and the need to stratify non-modifiable pre-existing risk factors in the older adult to measure the variable ^(^ 21 ^)^. 

The hypotheses of the theory produced in the operating system from the relationships between empirical indicators, when tested, will verify relationships between the concepts of the microtheory, estimating the validity and reliability of the empirical indicators in older adult in the ICU. The scores or values obtained in measurements of the hypothesis testing variables are defined as measurement units, including nominal and ordinal intervals and measurement ratio levels ^(^ 16 ^)^. 

The study has limitations circumscribed to its theoretical nature, and the microtheory has not yet been tested in older adult in the ICU. The construction procedures of the elements of the theoretical system are dependent on the state of knowledge and worldview of the main author who elaborated the theory in her doctorate. However, the analysis and evaluation of other researchers of the original theory material may have minimized this subjectivity. Also, the hypotheses have not yet been subjected to testing for refutation or maintenance, which does not represent a problem in itself, but a temporal condition for the development of the microtheory, for which it is recommended that further studies be carried out to validate theories.

MiTCare-DEP is innovative and unprecedented, as it provides model instruments for use in clinical nursing practice, providing identity to the profession. Furthermore, it contributes to the originality and originality of a Brazilian nursing microtheory, ready for use in clinical practice in care for the prevention of delirium in older adult in ICUs. The microtheory on the screen indicates the practical implementation of a set of focal care interventions, closely related to the particularities and integrality of the older adult, and a set of contextual care interventions, related to the specificities of intensive care.

Advances in scientific knowledge, since the developed theory directs nursing care so that interventions act as stimuli in the older adult to achieve the adaptive response to the prevention of delirium in the ICU. It is understood that this new perspective is necessary to enable a new look at older adult hospitalized in the ICU, with regard to the promotion of nursing care.

## Conclusion

MiTCare-DEP prescribes care “for” and “in” the prevention of *delirium,* which support nursing care for older adult in intensive care. It is considered that there is a dynamic, non-linear and complex interaction between contextual and focal stimuli, requiring high levels of organization of nursing care. The interaction between focal stimuli and contextual stimuli support that nursing care in the prevention of *delirium* in older adult in the ICU is formed by a set of multifactorial influences capable of stimulating the adaptive response to the prevention of *delirium* in the older adult in the ICU. 

Analogously to the theoretical inferences of the RAM constructs, this microtheory indicates the practical implementation of a set of focal care interventions, closely related to the particularities and integrality of the older adult, and a set of contextual care interventions, related to the specificities of intensive care. The developed microtheory directs nursing care so that these interventions act as stimuli in the older adult to achieve an adaptive response to prevention.
